# The Unique GGA Clathrin Adaptor of *Drosophila melanogaster* Is Not Essential

**DOI:** 10.1371/journal.pone.0045163

**Published:** 2012-09-20

**Authors:** Shan Luan, Anne M. Ilvarsonn, Joel C. Eissenberg

**Affiliations:** 1 Department of Biology, Macelwane Hall, Saint Louis University, St. Louis, Missouri, United States of America; 2 Edward A. Doisy Department of Biochemistry and Molecular Biology, Saint Louis, University School of Medicine, St. Louis, Missouri, United States of America; Institut Curie, France

## Abstract

The Golgi-localized, γ-ear-containing, ARF binding proteins (GGAs) are a highly conserved family of monomeric clathrin adaptor proteins implicated in clathrin-mediated protein sorting between the *trans*-Golgi network and endosomes. GGA RNAi knockdowns in Drosophila have resulted in conflicting data concerning whether the Drosophila GGA (dGGA) is essential. The goal of this study was to define the null phenotype for the unique Drosophila *GGA.* We describe two independently derived *dGGA* mutations. Neither allele expresses detectable dGGA protein. Homozygous and hemizygous flies with each allele are viable and fertile. In contrast to a previous report using RNAi knockdown, *GGA* mutant flies show no evidence of age-dependent retinal degeneration or cathepsin missorting. Our results demonstrate that several of the previous RNAi knockdown phenotypes were the result of off-target effects. However, GGA null flies are hypersensitive to dietary chloroquine and to starvation, implicating GGA in lysosomal function and autophagy.

## Introduction

Newly synthesized proteins destined for certain organelles or for secretion are trafficked to their destinations by membrane-bounded vesicles. Vesicle formation is initiated by the recruitment of clathrin, which imposes a curvature to membrane surfaces eventuating in vesicles. The formation of clathrin-coated vesicles (CCVs) depends on adaptor proteins (APs), which are recruited by GTP-ARFs on cellular membrane [1], [2]. However, details of cargo recognition and vesicle formation in endocytic and secretory sorting pathways remain elusive.

In 2000, five groups simultaneously identified Golgi-localized, γ-ear-containing, ARF binding proteins (GGAs) by yeast two-hybrid protein interaction with ARF3 [3] and by searching expressed sequence tag (EST) database for genes encoding proteins with VHS or γ-adaptin ear domain motifs homologous to those found in APs [4]–[7]. The structures of GGA family proteins consist of three folded domains: (i) the N-terminal VHS (Vps27, Hrs, STAM) domain, which recognizes the acidic dileucine motif of the cargo protein [7], [8], (ii) the GAT (GGA and TOM1) domain, which binds to GTP-ARF [3], [5], [9] and ubiquitin [10]–[12] and (iii) the C-terminal GAE (γ-adaptin ear) domain, which shares homology to the ‘ear’ domain of the γ-subunit of AP-1 and which interacts with accessory proteins [6], [13]–[16]. An unstructured hinge region connecting the GAT and GAE domains interacts with clathrin [13], [17], [18].

Although some phenotypes of GGA mutants have been established in yeast [5], [6], [18]–[20] and *C. elegans* [21], it is not yet clear whether GGA is essential in animals. Since GGAs are evolutionally conserved from yeast to mammals, we postulate GGA must either be essential for survival or indispensible under certain environmental conditions.

RNAi knockdown of single, two and three GGAs in mammalian cultured cells showed only mild missorting of Cathepsin D as well as memapsin 2 (BACE, membrane associated aspartic protease) accumulation in early endosomes [22]–[26], suggesting a modest contribution of mammalian GGAs to cellular homeostasis. However, a GGA2 knockout mutation in mice results in either early embryonic or neonatal lethality, depending on genetic background [14]. Mice that are doubly mutant for GGA1 and GGA3 show neonatal lethality, whereas mice singly mutant for either GGA1 or GGA3 have normal lifespans and fertility [27].

Drosophila has only one GGA protein. Compared to mammalian GGAs, Drosophila GGA (dGGA) is no more similar to any one of the mammalian GGAs, with identity ranging between 23% in GGA3 to 27.5% in GGA1 and with similarity ranging between 41% in GGA3 to 45% in GGA2. The difference between GGA2 and the other two mammalian GGAs is the absence of internal dileucine motif in GGA2, which is not subjected to phosphorylation and autoinhibition [28]. In this aspect, dGGA is similar to GGA2, since both lack an internal dileucine motif. dGGA recognizes the lysosomal enzyme receptor protein (LERP) [29], suggesting a role in protein sorting from the Golgi. dGGA knockdown by RNAi in Drosophila Schneider S2 cultured cells results in a 50% decrease in LERP processing [30], suggesting a defect in LERP sorting to lysosomes. In Dmel2 cells, knockdown of dGGA showed accumulation of GFP-tagged LERP in larger vesicles with total LERP protein level unchanged, which also suggests impaired LERP processing [25]. *In vivo* analysis of dGGA by RNAi knockdown showed conflicting results. Knocking down dGGA with an *Actin-Gal4* driver showed lethality and semi-lethality by two groups [31], [32]. The semi-lethal *dGGA-RNAi3* hairpin construct (from Vienna Drosophila RNAi Center, VDRC 3269 and 3270) [32] overlaps with lethal *dGGA-RNAi2* [32] and lethal *UAS-CG3002*/*dGGA-RNAi* (from National Institute of Genetics, Japan) [31]. A completely distinct hairpin construct, *dGGA-RNAi1*, showed mild semi-lethality but also did not knock down dGGA protein completely [32]. Using the same VDRC 3269 and 3270 constructs with *tubulin-Gal4* driver, Hirst and Carmichael reported that dGGA knockdown flies are viable and fertile with no apparent phenotype [33], albeit they still express low levels of dGGA (less than 5%) [33]. In addition, *dGGA-RNAi2* was also tested using *tubulin-Gal4* driver and it was lethal (Eissenberg unpublished data) Both *Actin-5C* and *tubulin* are housekeeping genes and are expressed in most or all tissues of the fly throughout development.

Taken together, the results from GGA knockout or knockdown in various model organisms have yielded conflicting results concerning an essential requirement for GGA family proteins. The potential for an off-target effect associated with RNAi lead us to question whether dGGA is essential in flies. Here, we describe the generation and characterization of two dGGA null mutant alleles, one generated by P-element excision and the other by targeted knockout using homologous recombination. Neither mutant makes detectable dGGA protein and flies carrying each are viable and fertile under normal laboratory culture conditions.

## Results

### Generation of a P-element Excision-mediated Allele of d*GGA*

Previous studies using RNA interference to knock down dGGA expression suggested that *dGGA* is essential, and implicated dGGA in sorting of lysosomal hydrolases and in preventing retinal degeneration [31], [32].

**Figure 1:**
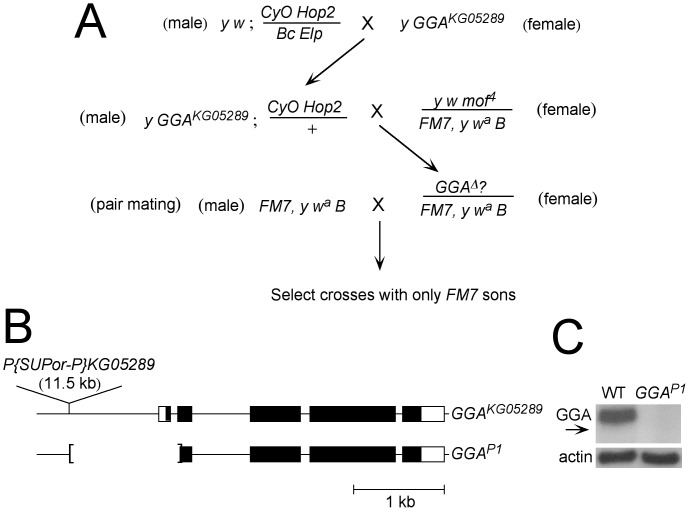
Generation of the *GGA^P1^* mutant allele. **A.** Scheme to generate candidate transposon excision-mediated deletions of GGA. **B.** Structures of the *GGA^KG05289^* and *GGA^P1^* alleles. **C.** Western blot showing GGA protein levels relative to cytoplasmic actin in wild type (WT) and *GGA^P1^* adult flies. Arrow indicates the predicted position of a truncated GGA^P1^ protein, assuming the first in-frame AUG codon is used.

To generate a deletion mutation at the *dGGA* locus, we took the approach of generating imprecise P-element excisions [34]. As a target for excision, we used the *GGA^KG05289^* allele, which carries a copy of the 11.5 kb transposon *P{SUPor-P}* inserted ca. 1 kb upstream of the *dGGA* transcription start site. We used a scheme (Figure 1A) in which the *P{SUPor-P}KG05289* element is mobilized by P-element transposase provided by crossing to a stock carrying the *Hop2* transposase source and scored for loss of the *y^+^* marker and X-linked recessive lethality among the progeny. Based on these criteria, we identified ten candidate lines for *dGGA* mutations from this screen.

Among these ten lines, we defined a deletion at the *dGGA* locus in one line, which we call *GGA^P1^*. This allele is the result of a deletion that removes 1117 bp upstream of the *dGGA* start codon and extends for 117 bp downstream from the start codon (Figure 1B). Thus, this deletion removes the promoter, transcription start site, 5′ untranslated region, start codon and 13 additional codons, together with the first intron.

### The *GGA^P1^* Allele is not Recessive Lethal

The screen from which the *GGA^P1^* allele was recovered was designed to select only *dGGA* mutations that are associated with recessive lethality. To test whether the lethality associated with the *GGA^P1^* chromosome maps to the *dGGA* locus, we tested whether a duplication of a region of the X chromosome containing GGA carried on the Y chromosome could complement the lethality associated with the *GGA^P1^* chromosome. *dGGA* is located within cytological region 8F1. Surprisingly, we found that *Dp(1;Y)BSC58*, which carries an X chromosome fragment with one breakpoint between 8D9 and 8E4 and the other at 9E1, fails to complement the lethality of the *GGA^P1^* chromosome. However, since the distal breakpoint in the duplication is close to the *dGGA* locus, and since the Y chromosome in Drosophila is heterochromatic in somatic tissue, we considered the possibility that the wild type *dGGA* allele carried on the translocated fragment might be silenced by heterochromatic position effect. Consistent with this hypothesis, we found that a different X chromosome fragment carried on the Y chromosome, *Dp(1;Y)BSC144,* does complement the lethality of the *GGA^P1^* chromosome.

Since the interval of X chromosome carried by *Dp(1;Y)BSC144* is relatively large (cytological interval 8A2–8F9; >500 kb), we used a nested set of truncated derivatives of *Dp(1;Y)BSC144* to refine the map position of the lethal mutation on the *GGA^P1^* chromosome (Table 1). Surprisingly, all the derivatives that included X chromosome material proximal to position 8C12 failed to complement the lethality, although all contain the *dGGA* locus. This demonstrated that the lethal mutation on the *GGA^P1^* chromosome is separable from the *GGA^P1^* mutation.

**Table 1:** Complementation mapping of lethal mutation associated with *GGA^P1^.*

Duplication	Cytologicalinterval	Complements *GGA^P1^*lethality
*Dp(1;Y)BSC58*	[8D9–8E4] –9E1	no
*Dp(1;Y)BSC144*	8A2–8F9	yes
*Tp(1;Y)BSC145*	[8A2–8B6] –8F9	yes
*Tp(1;Y)BSC147*	[8C3–8C4] –8F9	yes
*Tp(1;Y)BSC148*	[8C4–8C12] –8F9	no
*Tp(1;Y)BSC150*	[8D1–8D2] –8F9	no
*Tp(1;Y)BSC151*	[8D2–8D4] –8F9	no
*Tp(1;Y)BSC152*	[8D4–8D9] –8F9	no
*Tp(1;Y)BSC153*	[8D4–8D9] –8F9	no
*Tp(1;Y)BSC154*	[8D9–8E4] –8F9	no
*Tp(1;Y)BSC155*	[8E4–8E12] –8F9	no
*Tp(1;Y)BSC156*	[8E12–8F9] –8F9	no
*Dp(1;3)DC201*	8C1–8C4	no
*Dp(1;3)DC202*	8C1–8C8	no
*Dp(1;3)DC203*	8C4–8C13	yes

To further localize the lethal mutation on the *GGA^P1^* chromosome, we tested three third chromosome BAC transgenes with defined X chromosome fragments for complementation activity. Together, the three transgenes span the cytological interval 8C1–8C13 (Table 1). Neither *Dp(1;3)DC201* nor *Dp(1;3)DC202* complement the lethality, but *Dp(1;3)DC203* does. Taken together, these results place the *GGA^P1^* chromosome-linked lethal in the interval 8C8–8C12, and demonstrate that the *GGA^P1^* allele is not a recessive lethal mutation.

Intriguingly, the 8C8–8C12 interval contains the gene encoding the Drosophila AP-1 adaptin subunit AP-1γ. Previous studies suggested that dGGA is functionally redundant with the heterotetrameric clathrin adaptor AP-1 in the sorting of the lysosomal enzyme receptor LERP from the *trans*-Golgi into clathrin-coated vesicles in cultured cells [25], [30]. We considered the possibility that the lethality in the 8C8–8C12 interval that arose simultaneously in our screen with the *GGA^P1^* allele might be the result of a mutation in *AP-1γ*, and may reflect a synthetic lethal interaction between these two mutations. To test this hypothesis, we used a mutant with a PBac{RB} transposon insertion in the *AP-1γ* gene to test for complementation. This mutation is recessive lethal. Female flies heteroallelic for *GGA^P1^* and *PBac{RB}AP-1γ* are viable and fertile, demonstrating that the lethality of *GGA^P1^* is not due to an *AP-1*γ mutation.

The ability to rescue the recessive lethality of the *GGA^P1^* chromosome with *Dp(1;3)DC203* permits an assessment of dGGA protein expression in *GGA^P1^* mutant flies. No GGA protein is detectable by Western blot in *GGA^P1^*; *Dp(1;3)DC203* flies (Figure 1C) using antibody against dGGA (antibody epitope sequence amino acids 261[QLVADTL]- 660[end]) [25].

### *GGA^P1^* does not Cause Age-dependent Retinal Degeneration

**Figure 2:**
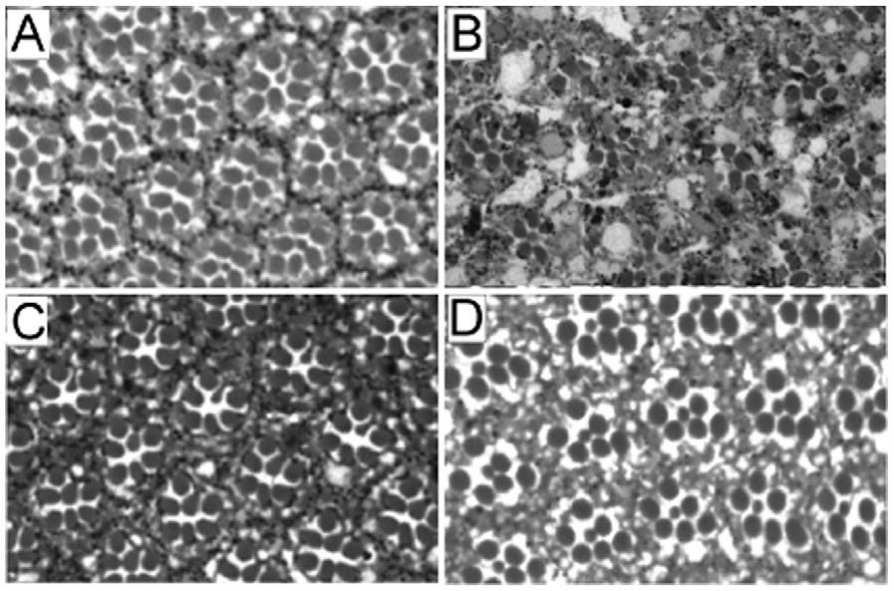
*GGA^P1^* does not cause age-dependent retinal degeneration. Wild type (**A**), *ey/GMR-Gal4>*; *dGGA RNAi2* (**B**), *GGA^P1^*; *Dp(1;3)DC203*;(**C**) and *w^1118^*; *Dp(1;3)DC203* (**D**) adult flies were aged 14 d. at 25°C, and the eyes sectioned, stained with toluidine blue and examined by light microscopy. Ommatidial organization and rhabdomere number/morphology are normal in panels **A**, **C** and **D**, but highly degenerated in panel **B**. Note the small number of pigment granules in the *w^1118^*; *Dp(1;3)DC203* eyes reflects the weak *white* transgene expression in this stock relative to the wild-type *white* gene expression in the Oregon R and *GGA^P1^*; *Dp(1;3)DC203* stocks.

RNAi knockdown of dGGA using the *dGGA-RNAi2* hairpin under eye-specific Gal4 drivers results in age-dependent retinal degeneration [32](Fig. 2B). To test whether a comparable degeneration is associated with the *GGA^P1^* mutation, we examined sectioned compound eyes from two-week-old *GGA^P1^*; *Dp(1;3)DC203* adults (Fig. 2C). When compared with comparably aged wild type (Oregon R; Fig. 2A) and *w^1118^*; *Dp(1;3)DC203* controls (Fig. 2D), we found no significant difference in ommatidial array organization or rhabdomere number/integrity.

### *GGA^P1^* does not Cause age-dependent Retinal Degeneration

RNAi knockdown of GGA using the *GGA-RNAi2* hairpin under eye-specific Gal4 drivers results in age-dependent retinal degeneration [32]. To test whether a comparable degeneration is associated with the *GGA^P1^* mutation, we examined sectioned compound eyes from two-week-old *GGA^P1^*; *Dp(1;3)DC203* adults. When compared with comparably aged wild type (Oregon R) and *w^1118^*; *Dp(1;3)DC203* controls, we found no significant difference in ommatidial array organization or rhabdomere number/integrity (Figure 2).

### Generation of a Targeted Knockout Allele of *dGGA*

The lack of recessive lethality and absence of age-dependent degeneration in *GGA^P1^* flies contrast sharply with phenotypes reported for dGGA RNAi knockdown [31], [32]. Since the *GGA^P1^* deletion only removes 13 codons and the next in-frame methionine codon is 16 codons downstream from the break point, it is formally possible that sequences upstream from the deletion contain a cryptic promoter and transcription start site. If this were the case, mRNA could potentially be translated using that downstream methioninyl codon as a start codon, resulting in a slightly truncated dGGA protein that still includes the VHS domain. Thus, we cannot confidently exclude the possibility that a small amount of functional dGGA, undetectable by Western blot analysis, could be expressed from the *GGA^P1^* allele, and that this allele is not a functional null mutation.

**Figure 3:**
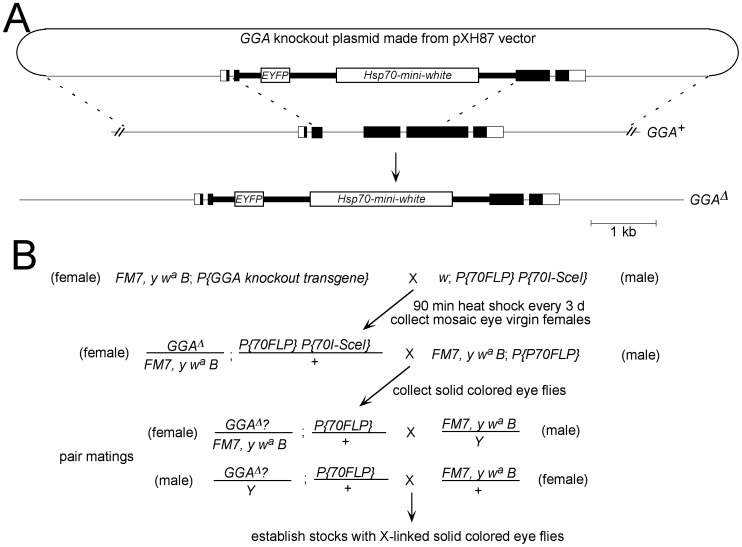
Generation of the *GGA* knockout flies. **A.** Molecular basis for *GGA* targeting. The *GGA* knockout plasmid was created by cloning ∼3 kb GGA upstream and downstream sequences into multiple cloning sites of the pXH87 vector [35]. FLP recombinase catalyzes the excision of the knockout cassette and I-SceI cleavage releases a DNA fragment from the excised circle, which then can undergo homologous recombination with the targeted genomic DNA sequence to generate *GGA^Δ^*. For simplicity, the representations of the two genes to the right of *GGA* are omitted. **B.** Scheme used to generate candidate *GGA^Δ^* flies.

**Figure 4:**
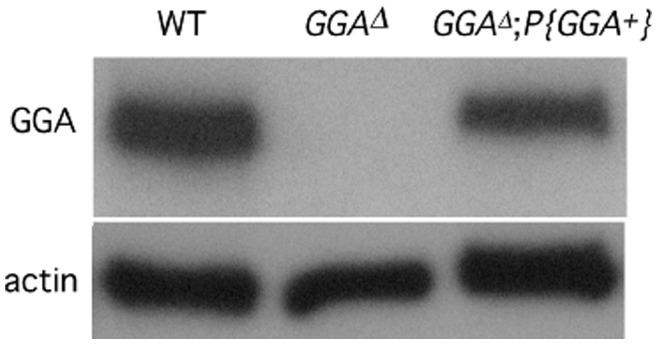
A GGA transgene complements the protein-null phenotype of *GGA^Δ^* flies. *GGA^Δ^;P{GGA^+^}* restored the GGA protein to wild type levels. Actin levels from corresponding lane are shown below. All images were taken from the same blot at the same exposure.

To generate a *dGGA* null mutation, we turned to site-directed mutagenesis using an ends-out gene replacement strategy adapted from Chen et al. (2009). A 3 kb fragment extending 5′ from the *dGGA* coding region and a 2.7 kb fragment extending 3′ from the *dGGA* coding region were separately amplified by PCR and cloned into the pXH87 vector [35] (Figure 3A). We included the *FM7* balancer chromosome during the knockout mobilization steps in order to (1) rescue the *dGGA* null mutant flies in case knocking out *GGA* causes lethality and (2) increase the efficiency of recovering targeted events on the X chromosome [35] We used a scheme (Figure 3B) in which the *p{GGA knockout transgene}* targeting cassette is mobilized by FLP recombinase and linearized by I-SceI provided by *P{70 FLP} P{70-Sce-I}* after heat shock. We collected ∼3000 mosaic- or white-eyed virgin females and mass-crossed these with males of a stock carrying *P{70 FLP}* to eliminate any residual autosomal copies of *p{GGA knockout transgene}*. From the progeny, 300 flies carried the *w* ^+^ transgene marker and seven of these carried the transgene marker linked to the X chromosome. Using PCR primers flanking outside the targeted region and inside the *EYFP*-mini-*white* knockout cassette, as well as Western blot analysis, we were able to confirm six lines in which *GGA* was knocked out (Figure 4 and data not shown). The targeting rate was calculated as 0.23%, which is at the low end of the targeting frequency range reported by Chen et al. (2009) for autosomal knockouts using this approach. *GGA^Δ^* was the result of replacing a total of 1131 bp, including 536 bp of the third exon, the entire third intron and 526 bp of the fourth exon, with the *EYFP* and *mini-white* cassette of pXH87. This results in the loss of 353 codons, including the C-terminal half of the VHS domain, the entire GAT domain and the N-terminal half of the hinge domain. All the six *GGA^Δ^* lines are viable and fertile. Like *GGA^P1^*, *GGA^Δ^* flies show no evidence of age-dependent retinal degeneration at 2 weeks (data not shown).

To test for possible semi-lethality associated with *dGGA* mutations, we compared the ratio of heterozygous *dGGA* mutant daughters to *dGGA* sons generated from a cross of *dGGA* mutant females and wild-type male flies with the ratio of heterozygous *dGGA* mutant daughters to *GGA* ^+^ sons recovered from the reciprocal control cross. Statistical analysis suggested there is no selective mortality among dGGA mutants relative to controls for either the *GGA^P1^* or *GGA^Δ^* allele (Table 2).

**Table 2:** Testing semi-lethality of the *GGA^Δ^ and GGA^P1^* alleles.

Crosses	F1 female	F1 male	p-Value
female *GGA^Δ^* X male *yw*	229	183	0.5310
female *yw* X male *GGA^Δ^*	223	195	
female *GGA^P1^* X male *yw*	192	174	0.7815
female *yw* X male *GGA^P1^*	259	225	

### *dGGA* Knockout Mutation Fails to Rescue RNAi Knockdown of dGGA

The absence of detectable lethality associated with either of the *dGGA* mutations we isolated raises the possibility that previously reported lethality associated with dGGA RNAi knockdowns could be off-target. Previous research showed that the *GGA-RNAi2* hairpin is lethal in combination with the *Actin5C-Gal4* driver, with the lethal period in late pupation [32]. Another GGA hairpin construct available from the Vienna Drosophila Resource Center (VDRC) is reportedly lethal [31] or semi-lethal [32] with the *Actin5C-Gal4* driver. Since the sequences targeted by both hairpins are completely deleted from the *GGA^Δ^* allele, combining the *GGA^Δ^* allele with such hairpins provides an explicit test of whether the RNAi-mediated lethality is on- or off-target. Since the VDRC hairpin sequence (construct ID 1710) is entirely contained within the *GGA RNAi2* hairpin sequence, we generated mutant flies that were *GGA^Δ^; GGA-RNAi2* and crossed females of this stock with males of a stock containing *Act5C-Gal4* driver. We recovered no driver-containing sons, demonstrating that the lethality associated with the *GGA-RNAi_2* and 1710 hairpins must be off-target.

### Defining the Minimal *dGGA* Gene

The sequence location of *GGA* gene is predicted to span the region between X: 9,495,067 and 9,498,313 according to flybase.org. *Hex-A*, which spans the region between X: 9,479,918 and 9,482,434, is the closest annotated gene upstream of *dGGA*. Thus, the transcriptional control of *dGGA* could be regulated by sequences anywhere in the 12.6 kb interval between these genes. However, *dGGA* lacks a TATA box, suggesting that it could be a housekeeping gene [36]. We therefore inferred that most of *dGGA*’s regulatory elements are very close to the transcription start site. Since the 3′ UTR of *dGGA* overlaps with the 3′ UTR of the downstream gene *CG3004*, we hypothesize that the functional *dGGA* gene ends at the annotated transcription termination site X: 9,498,313. To define the minimum genomic sequence necessary to support *dGGA* expression, we cloned *dGGA* genomic sequences including 1 kb upstream of the *dGGA* transcription start site and inserted this sequence as a transgene in transgenic flies *yw*; *P{GGA^+^}*. To test for autonomous expression of this transgene, we crossed autosomal inserts into either a *GGA^P1^* or *GGA^Δ^* background. Western blot analysis showed that this transgene supports GGA expression at or near wild-type levels (Figure 4 and data not shown).

### *GGA^P1^* and *GGA^Δ^* Flies have No Defect in Cathepsin L or Cathepsin D Processing

**Figure 5:**
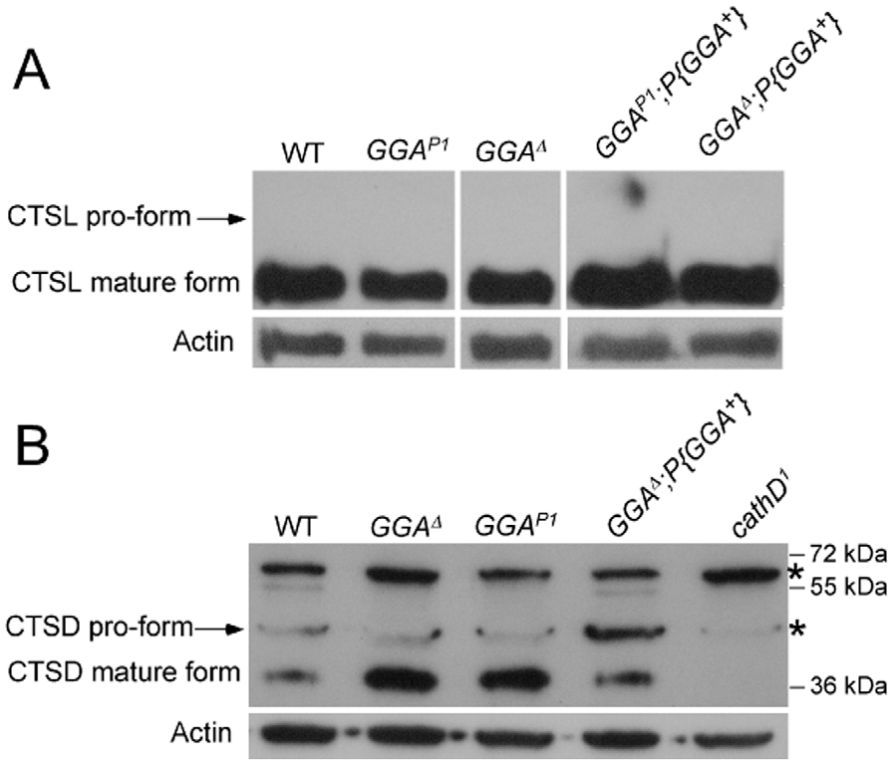
Cathepsin L and cathepsin D processing and steady-state levels are not impaired in GGA null flies. A. Western blot showing mature CTSL protein level relative to cytoplasmic actin in adult wild-type (WT), *GGA^P1^, GGA^Δ^, GGA^P1^;P{GGA^+^}* and *GGA^Δ^;P{GGA^+^}* rescue flies. The unprocessed form of CTSL is undetectable across all the samples. Actin levels from corresponding samples are shown below. **B.** Western blot showing mature CTSD protein level relative to cytoplasmic actin in adult wild-type (WT), *GGA^Δ^, GGA^P1^, GGA^Δ^;P{GGA^+^}* and cathepsin D null mutant flies (*cathD^1^*) [37]. The CTSD pro-form comigrates with a cross-reactive antigen also present in CTSD null mutant (asterisk). An antigen of ca. 63 kDa also appears in all samples, but since this antigen is also present in *cathD^1^* flies (asterisk), it is unrelated to CTSD. Actin levels from corresponding lanes are shown below. All images using a given antibody were taken from the same blot at the same exposure.

GGA family proteins have previously been implicated in the sorting and/or processing of lysosomal hydrolases [29]. Missorting of the cathepsin L and cathepsin D proform was reported for dGGA RNAi knockdowns [32]. We examined cathepsin L and cathepsin D sorting and processing in adult flies. Western blot analysis of extracts from *GGA^P1^* and *GGA^Δ^* flies showed no significant defect in either cathepsin L and cathepsin D sorting or processing (Figure 5). In the case of cathepsin D, we cannot exclude the possibility that a minor amount of pro-form accumulates in the knockout flies, since a weakly cross-reacting antigen appears in the position of the proform in all extracts, including extract from a cathepsin D null stock [37]. Larval hemolymph showed no increase in accumulation of the pro-cathepsin L (data not shown). Thus, the defects in cathepsin sorting reported for *dGGA* RNAi knockdown [32], like the lethality associated with dGGA RNAi knockdown, appears to be an off-target effect. dGGA does not have a significant sorting or processing effect on cathepsin L and cathepsin D. However, these results do not exclude defects in sorting and/or processing of other lysosomal hydrolases.

### Lysosome Proteolytic Function is Unaffected in *GGA^P1^* and *GGA^Δ^* Flies

Previously, impaired lysosomal function was reported in dGGA RNAi knockdown flies, with accumulation of a LAMP1-GFP fusion protein in vesicles [32]. The LAMP1-GFP fusion protein is trafficked to lysosomes in Drosophila and places GFP on the luminal face of vesicle membrane, resulting in rapid extinction of GFP fluorescence upon fusion with lysosomes [38], [39]. We examined the lysosomal proteolytic function of GGA knockout third instar larval by fluorescent microscopy of salivary glands expressing the LAMP1-GFP fusion protein. Compared to the control GFP-LAMP1 expression, there is no apparent increase in of the numbers or size of fluorescent vesicles in mutant flies that would be indicative of GFP stabilization in defective lysosomes (data not shown). By this criterion, then, the lysosomal proteolytic function in *GGA^P1^* and *GGA^Δ^* flies seems to be normal. Effects on other classes of lysosomal hydrolases, however, are not ruled out by this assay.

### *GGA^Δ^* Flies are Hypersensitive to Dietary Chloroquine

**Figure 6:**
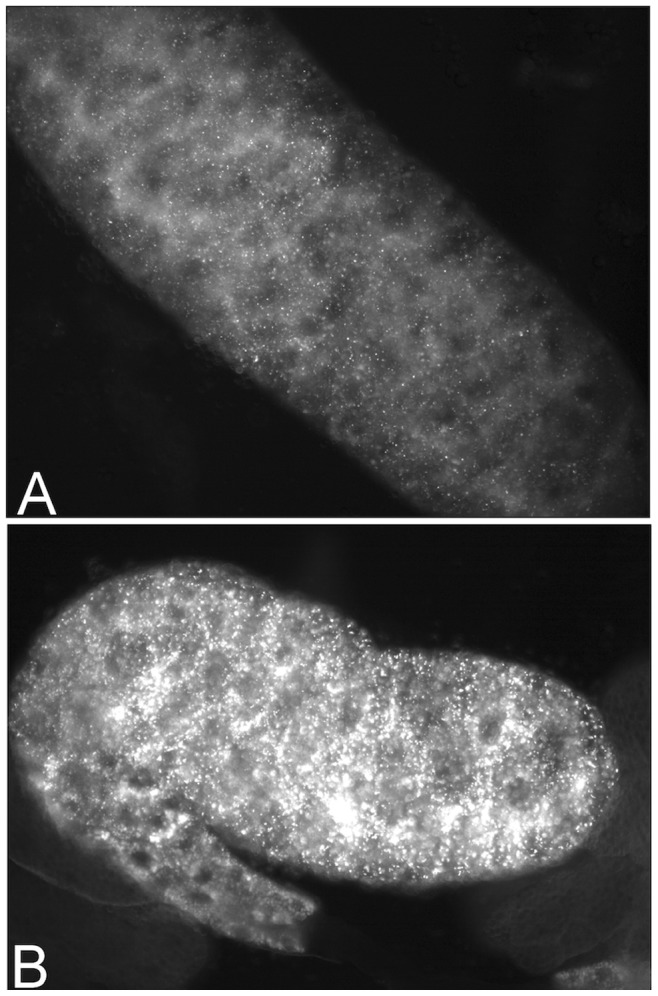
Dietary chloroquine impairs lysosome-mediated proteolysis. Salivary glands were dissected from LAMP1-GFP-expressing third instar larvae that were fed for 36 hr on instant Drosophila food reconstituted with water (A) or 10 mM chloroquine (B). Increased GFP fluorescence in the salivary glands of chloroquine-fed larvae results from both larger and brighter fluorescent vesicles.

As dGGA is implicated in sorting hydrolytic enzymes from the *trans*-Golgi network to lysosomes, we hypothesized that if the lysosome function were impaired by environmental factors, additional lysosomal defects due to loss of dGGA would enhance the lysosomal impairment. To test this hypothesis, we tested whether loss of dGGA enhances sensitivity to dietary chloroquine. Chloroquine is a drug used in malaria treatment that raises lysosomal pH, and impairs lysosomal enzyme activities [40], [41]. To establish that dietary chloroquine impairs lysosome function, we fed third instar larvae expressing LAMP1-GFP on food containing 10 mM chloroquine for 36 hours. We examined the lysosomal function of larval salivary glands by fluorescent microscopy. There is a consistent increase in accumulation of GFP-LAMP1 in treated larvae compared to controls (Figure 6), testifying to impaired lysosomal proteolysis in chloroquine-fed larvae.

**Figure 7:**
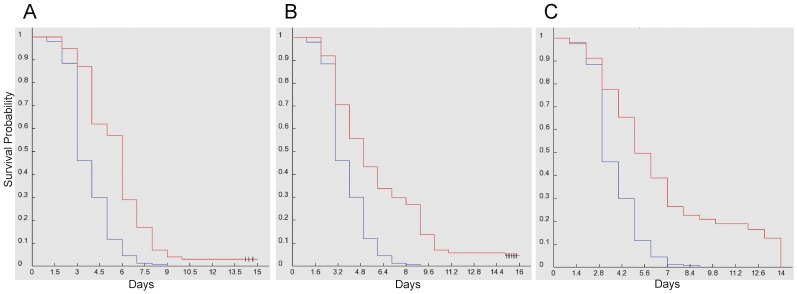
GGA mutant flies are hypersensitive to dietary chloroquine. Blue lines represent: *GGA* ^Δ^ males (n = 181 and median survival time 2.90 days) Red lines represent **a.** *yw* males (n = 97, P-value <0.0001 and median survival time 5.25 days) **b.** *yw; P{ GGA* ^Δ^ *} ^A2^* males (n = 151, P-value <0.0001 and median survival time 4.45 days) **c.** *yw; P{ GGA* ^Δ^ *} ^E2^* males (n = 161, P-value <0.0001 and median survival time 4.98 days).

Dietary chloroquine at or above 10 mM causes significant toxicity in Drosophila adults (our unpublished observations). If dGGA is required for lyososomal homeostasis, *GGA^Δ^* flies should be hypersensitive to dietary chloroquine. Newly eclosed adult flies raised on standard food were placed on food supplemented with 20 mM chloroquine. To minimize the effect due to background genotype, we tested sons from crosses of female *GGA^Δ^* and male *yw* parents and used sons from the reciprocal cross as controls. As an additional control, sons from the cross of female *GGA^Δ^* and male *yw;P{GGA+}* were used to test the ability of a wild-type dGGA transgene to complement the knockout phenotype. The median survival time for *GGA^Δ^* males was 2.9 days, for control *yw* males was 5.25 days, and for the two rescue lines *GGA^Δ^; P{GGA^+^}^E2^* and *GGA^Δ^; P{GGA^+^}^A2^* were 4.98 days and 4.45 days, respectively (Figure 7). *GGA^Δ^* survival time is significantly reduced from both the control and rescue crosses with P-values <0.0001. The hypersensitivity of *GGA^Δ^* mutants to chloroquine is consistent with a role for dGGA in lysosomal homeostasis.

### dGGA Mutations Interact with the Autophagy-associated Gene *Blue cheese (Bchs)*

**Figure 8:**
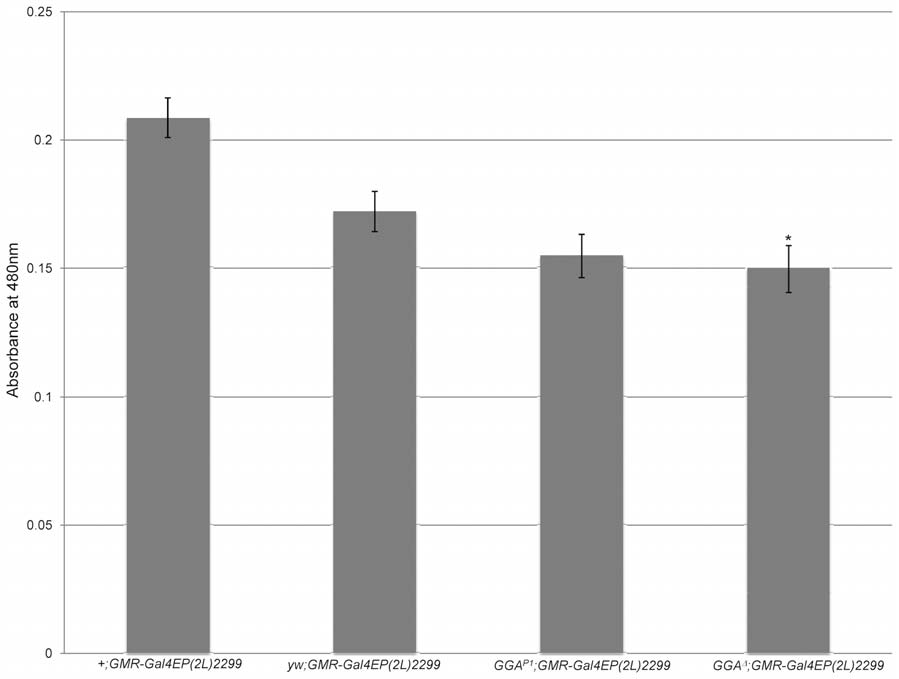
GGA knockout enhances the eye phenotype caused by *Bchs* overexpression. Control: *Bchs* overexpressing male *+; GMR-Gal4 EP(2L)2299, yw; GMR-Gal4 EP(2L)2299. Bchs* overexpressing male knocked down or out for GGA: *GGA^P1^; GMR-Gal4 EP(2L)2299* and *GGA* ^Δ^ *; GMR-Gal4 EP(2L)2299.* Amount of extracted red eye pigment was measured at 480 nm. (*)*GGA* ^Δ^ *; GMR-Gal4 EP(2L)2299* showed significant reduction in red eye pigment compared with both *+; GMR-Gal4 EP(2L)2299* and *yw; GMR-Gal4 EP(2L)2299* (P-value < 0.0008 and 0.04). *GGA^P1^; GMR-Gal4 EP(2L)2299* showed only significant reduction in red eye pigment compared with *+; GMR-Gal4 EP(2L)2299* (P-value < 0.0003).

Drosophila *Bchs* is the homolog of the human *Alfy* gene. Both gene products contain the highly conserved BEACH domain, FYVE zinc-finger domain and WD40 repeats [42]. The structure of Bchs suggests its function as a scaffolding protein in autophagic membrane trafficking [42], [43]. Overexpression of *Bchs* under an eye-specific driver leads to a reduced and rough eye phenotype in flies, a phenotype that is modified by mutations in genes encoding factors implicated in autophagy [44], [45]. When dGGA was knocked down in the eye by RNAi in the *Bchs* overexpression background, the rough eye phenotype was enhanced and the eye size further reduced, suggesting dGGA plays a role in autophagy [32]. We tested our GGA mutant lines in this assay to confirm a role for GGA in autophagy. To accurately quantify the differences of eye size between *Bchs* overexpression in *dGGA* mutant flies and wild-type flies, we measured the amount of red eye pigment in each genotype as an index of total eye volume. Both *GGA^P1^; GMR-Gal4 EP(2L)2299* and *GGA* ^Δ^ *; GMR-Gal4 EP(2L)2299* had less eye pigment in comparison with *+; GMR-Gal4 EP(2L)2299* (P-value <0.001). However, when compared *with yw; GMR-Gal4 EP(2L)2299* flies, only *GGA* ^Δ^ *; GMR-Gal4 EP(2L)2299* had a significant reduction (P-value <0.05) (Figure 8). It is formally possible that the *GGA^P1^* expresses a small amount of functional GGA, since the coding region is mostly intact. The significant eye pigment reduction in *GGA* ^Δ^ with *Bchs* overexpression, however, suggests that dGGA is a modifier for *Bchs* overexpression, thus implicating GGA in autophagy.

### *GGA^Δ^* Flies are Hypersensitive to Amino Acid Starvation

**Figure 9:**
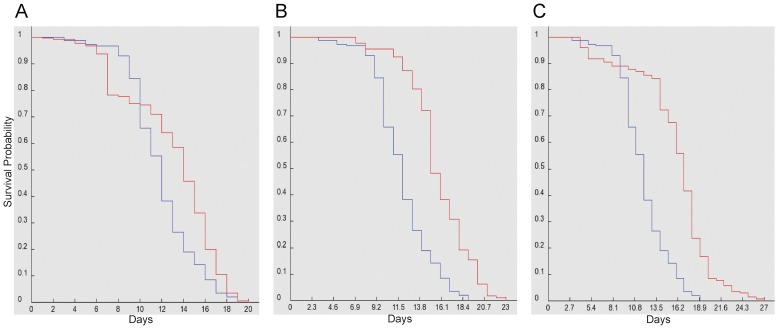
GGA mutant flies are hypersensitive to amino acid starvation. Blue lines represent: *GGA* ^Δ^ males (n = 212 and median survival time 11.30 days) Red lines represent **a.** *yw* males (n = 202, P-value <0.0001 and median survival time 13.65 days) **b.** *yw GGA* ^Δ^ *; P{GGA^+^} ^A2^* males (n = 131, P-value <0.0001 and median survival time 14.92 days) **c.** *yw GGA* ^Δ^ *; P{GGA^+^} ^E2^* males (n = 144, P-value <0.0001 and median survival time 16.4 days).

In starvation–induced autophagy, intracellular proteins and organelles are cannibalized to meet the minimum nutrient requirements of the starving cells. Autophagy is mediated by the lysosomal degradation pathway [46]. The enhancement of the *Bchs* overexpression phenotype by dGGA mutation suggests a role for dGGA in autophagy. We hypothesized that if dGGA were required for efficient autophagy, lack of dGGA would sensitize flies to starvation. In order to trigger autophagy, we placed newly emerged flies on amino acid deficient media [47], [48]. Crosses were performed as in the previous chloroquine experiment to minimize genetic background effects. During starvation, the median survival time for *GGA^Δ^* males was 11.29 days, while the median survival times for the control *yw* males was 14.03 days, and two rescue lines *GGA^Δ^; P{GGA^+^}^E2^* and *GGA^Δ^; P{GGA^+^}^A2^* had median survival times of 15.74 days and 14.50 days, respectively (Figure 9). The *GGA^Δ^* survival time is significantly different from the control cross and rescue cross with P-values <0.0001. The reduced survival of the *GGA^Δ^* mutant stock further implicates dGGA in autophagy.

## Discussion

Our understanding of GGA family protein functions in animals has increased significantly since the discovery of this family of monomeric clathrin adaptors in 2000. However, most studies were based on RNAi knockdowns in cultured cells or animal models, which can be complicated by potential off-target effect as well as incomplete protein elimination. Therefore, null mutations are required to define the true function of GGAs in multicellular organisms. Recently, the first GGA knockouts in animals were documented in mice by Govero, et al. (2012). Mutant phenotypes vary among the three murine GGAs and are affected by the different genetic background of those mutants [27]. Furthermore, other than small size and perinatal hypoglycemia, the phenotypes were uninformative as to the basis for lethality of GGA2 mutant mice or GGA1/GGA3 double mutant mice. The role of GGA family proteins in mammals thus remains elusive. The presence of a single GGA homolog in Drosophila provides a unique opportunity to assess the requirements for GGA in a metazoan.

In two independent screens, we generated two distinct gene knockout mutations. A key finding of this study is that both mutations, *GGA^P1^* and *GGA^Δ^,* showed no defect in survival, demonstrating that dGGA is not essential in normal development under laboratory conditions. Together with a functional dGGA transgene, we created a genetic tool kit for future GGA protein function research in Drosophila. For example, function of each domain could be analyzed by mutating each domain of dGGA and testing the mutations for function in transgenic flies carrying the *GGA^Δ^* allele. Although the method of targeted knockout was adapted from Chen et al. (2009), to our knowledge, this is the first report of a targeted knockout of an X-linked gene by this method.

Our results clearly demonstrate that GGA is not essential in Drosophila and agree with Hirst et al. (2011), who reported that *dGGA* knockdown flies develop normally with no obvious phenotype. In contrast, both Eissenberg et al. (2011) and Kametaka et al. (2012) reported lethality in RNAi knockdowns flies, leading us to speculate that the lethality in these cases was the result of an off-target effect of RNAi. We confirmed this hypothesis by showing that RNAi-induced lethality could not be complemented by *GGA^Δ^*, which lacks the RNAi-targeted sequences. In the absence of the targeted dGGA sequences, lethality must be due to an off-target effect of RNAi. We found a 19-nucleotide-long perfect match shared by all three hairpins that are reported to show lethality or semi-lethality and a sequence in the coding region of the *Arginine methyltransferase 4* (*Art4*) gene. However, Western blot analysis of extracts from the *dGGA-RNAi2* knockdown larvae probed for Art4 protein showed no significant reduction (data not shown). Thus, the basis for the off-target effect remains unknown.

Previous research implicated GGA family proteins in the sorting of lysosomal hydrolases (cathepsin B, D and L) [22]–[25], [29], [31], [32]. In *dGGA* knockout flies, there is no detectable defect in cathepsin L or cathepsin D processing, indicating that the missorting of cathepsin L and cathepsin D reported in dGGA knockdowns is likely to be an off-target effect generated by the RNA hairpin construct. The absence of a cathepsin sorting defect in *dGGA*-null flies is consistent with the evidence that dGGA is functionally redundant to AP-1 in cathepsin sorting in cultured cells [25]. AP-1 could substitute for the function of dGGA in lysosomal processing in GGA null mutants. In addition, the normal lysosomal proteolysis function of *GGA^P1^* and *GGA^Δ^* flies also suggests the previously reported stabilization of LAMP1-GFP in the cytoplasm of dGGA RNAi knockdown cells is off-target. The lack of a role for GGA in LAMP1-GFP turnover is also consistent with the functional redundancy of dGGA and AP-1.

We found no evidence for age-dependent retinal degeneration in *GGA^P1^* or *GGA^Δ^* flies as reported in *dGGA-RNAi2* knockdowns driven by *GMR-Gal4* [32]. Although dGGA is enriched in Drosophila testes [33], male flies hemizygous for either *GGA^P1^* or *GGA^Δ^* show no evidence of impaired fertility.

Under standard laboratory conditions, *dGGA* knockout mutants have no obvious apparent phenotype; the external cuticle and behavior appear normal. However, several reports have found GGA family proteins to be involved in sorting of lysosomal hydrolases, that are important for normal lysosomal function [22]–[25], [29], [31], [32]. This could be explained if the heterotetrameric clathrin adaptor AP-1 functions redundantly with GGA under standard lab conditions. A requirement for GGA is disclosed by hypersensitivity of our GGA knockout flies to dietary chloroquine, a lysosomal alkalinizing agent, supporting a model in which GGA family proteins contribute to lysosomal homeostasis under stress conditions.

Knocking out *GGA* in *Bchs* overexpression flies enhanced the eye phenotype, similar to results that was reported in *dGGA-RNAi2* knockdowns [32], suggesting that dGGA could directly or indirectly be involved in the process of autophagy. The role of dGGA in autophagy could be unique and distinct from dGGA and AP-1 function in lysosomal hydrolase sorting. This hypothesis is further supported by the observation that *GGA^Δ^* flies are hypersensitive to amino acid starvation, consistent with impaired autophagy. Since lysosomes are the final destination in autophagy, defects that alter lysosomal composition could lead to a lysosomal storage disorder, resulting in impaired autophagy and shortened life-span [49].

Our proposed link between lysosomal stress, autophagy and GGA in Drosophila suggests a possible explanation for the puzzling results of GGA knockdowns in mice. *Gga1/3* double mutant mice have a 47% death rate within one day after birth [27]. Depending on the genetic background, *GGA2* mutant mice have a death rate of 73% within two days after birth or die before E9 embryonic stage (earliest testable stage) [27]. We suggest that these lethal periods could be explained by the partial impairment of autophagy due to mutation in GGAs. In placental mammals, autophagy is crucial in specific developmental stages. Between the first cell division and implantation, autophagy is dramatically induced in cleavage stage mouse zygotes [50]. Atg5 (autophagy-related 5) knockout mice show early embryonic death [51]. Early zygotic death is also seen in one of the *GGA2* knockout mice lines. In addition, autophagy is induced after birth and returns to basal level in 1–2 days [52]. This latter period of autophagic stress could explain the lethality within either 24 or 48 hours in *Gga1/3* double mutation and one of the *GGA2* mutant strains. The fact that mice lacking the α/β subunits of *N*-acetylglucosamine-1-phosphotransferase [the enzyme required for the first step in synthesis of the mannose-6-phosphate (M6P) sorting marker] are fully viable argues against a simple model in which defective sorting of lysosomal hydrolases alone causes stage specific death at periods of high autophagy [53]. It may well be that GGAs contribute to lysosomal function independently of their role in M6P receptor recognition. Alternatively, GGAs could promote apoptosis through a mechanism independent of lysosome hydrolase sorting. It would be interesting to look for molecular signatures of autophagy and/or autophagic cell death in the dead or dying mice to determine whether the link between autophagy and GGA suggested by the Drosophila data is conserved in mammals.

## Materials and Methods

### Drosophila Stocks

The following stocks were obtained from the Bloomington Drosophila Stock Center: *y GGA^KG05289^; Dp(1;Y)BSC58; Dp(1;Y)BSC144; Tp(1;Y)BSC145; Tp(1;Y)BSC147; Tp(1;Y)BSC148; Tp(1;Y)BSC150; Tp(1;Y)BSC151; Tp(1;Y)BSC152; Tp(1;Y)BSC153; Tp(1;Y)BSC154; Tp(1;Y)BSC155; Tp(1;Y)BSC156; Dp(1;3)DC201; Dp(1;3)DC202; Dp(1;3)DC203; y w; [70FLP][70I-SceI]/TM6, w; [70FLP]* and *w^1118^ PBac{RB}AP-1γ^304546^/FM7c*. The *y w; CyO Hop2/Bc Elp* stock was obtained from Dr. D. Dorsett. The *y w mof^4^/FM7, y w^a^ B* stock was obtained from Dr. J.C. Lucchesi. The *GMR-Gal4 EP(2L)2299* stock was obtained from Dr. K. Finley. The *yw; LAMP1-GFP* stock was obtained from Dr H. Krämer. Cathepsin D mutant stock (*cathD^1^*) was obtained from Dr M. Feany.

### Methods for *dGGA* Knockout

About 3 kb upstream of dGGA (using primers forward: 5′ attatatGGTACC-TTCGCCAACGGTAACGGTAAG 3′ Reverse: 5′ attatatACCGGT-AATTTGGACGCCTCGTCCTGGA 3′) and 2.7kb downstream of GGA (using primers forward: 5′ attatatGCATGC-GGATCGGATGCCCAGCTTCA 3′ reverse: 5′ attatatCTCGAG-TTACCAGCACCGCGCCA CTTAT 3′) were amplified from genomic DNA and cloned into the pXH87multiple cloning sites on either side of the *EYFP* and *mini-white* cassette [35]. Plasmid DNA was sent to BestGene Inc (Chino Hills, CA) to generate transgenic flies and transgenic flies were selected based on eye pigmentation conferred by the *p{GGA knockout}* transgene. For the knockout strategy (Fig. 3), we started with 2000 virgin females in 100 vials and crossed these with *w;P{70FLP}P{70I-SceI}* males. After 3 days, the flies were transferred to new vials and the progeny in the first vial were heat shocked at 38°C for 90 min. 3000 mosaic or white eyed virgin females were recovered from the initial crosses and these were mated with *P{70FLP}* males. 300 non-mosaic colored eyed flies were recovered from this cross and these were pair-mated to *FM7, y w^a^ B* flies. By crossing with *FM7, y w^a^ B* flies, we could determine which progeny carried an X-linked insertion of the knockout cassette. PCR analysis was used to confirm the site of insertion at the *GGA* locus. Primers are:

Forward covers 3 kb fragment: 5′ GCAATGGGCTATTCTGGGTATCAC 3′.

Reverse covers 3 kb fragment: 5′ TGCTCAGGGCGGACTGGTAG 3′.

Forward covers 2.7 kb fragment: 5′ GGCAAGGTCATCCTGGAGACG 3′.

Reverse covers 2.7 kb fragment: 5′ TCCCGGACTAATCCCAGTTGCT 3′.

### dGGA Genomic Transgene Construct

Using primer pairs forward 5′ TCTAGA AACAGTTTTAGCCAGACCGATTGA 3′ and reverse 5′ TCTAGA GAAACAGTGGGGAGAGTAGCAATG 3′, 4.5 kb of *GGA* genomic DNA was generated. The GGA fragment, spanning 1 kb upstream of the transcription start site to just beyond the polyadenylation site (X:9496311, 9498217) was cloned in to *P{CaSpeR}*. Transgenic flies were generated by BestGene Inc (Chino Hills, CA). Transformants were identified based on eye pigmentation conferred by the *P{CaSpeR-GGA^+^}* transgene.

### Drosophila Stockkeeping

All crosses, except where noted, were maintained on standard cornmeal-agar-molasses medium at 25°C.

### Drosophila Eye Sections

Adult Drosophila heads were removed from bodies and the antennae removed to facilitate fixation. Tissue pieces were fixed in 3.5% glutaraldehyde in 0.1 M sodium cacodylate buffer (pH 7.25) containing 5% sucrose and 2 mM calcium chloride for at least for 3 d. at 4°C. The tissue was then washed 2–3×15 min in 0.1 M sodium cacodylate buffer containing 5% sucrose (this and all subsequent steps up to polymerization were at room temperature) and post-fixed in 1% osmium tetroxide in 0.1 M sodium cacodylate buffer containing 5% sucrose for 3–4 hours at RT on a rotator. The tissue was then washed 2 x 10 min in distilled water, dehydrated in 2 x 10 min steps through 35%, 50%, 75%, 95% and 100% ethanol, washed 2 x 10 min in propylene oxide and infiltrated with a 1∶1 mixture of Embed resin (Polysciences, Inc., Warrington, PA) and propylene oxide overnight on a rotator. The tissue was then incubated in fresh Embed resin for 6 hours, transferred to BEEM capsules filled with fresh resin, and polymerized overnight at 70°C. 0.5 µm thick sections were cut using a glass knife on a Reichert Ultracut E ultramicrotome (Depew, NY), collected and heat-attached to glass slides, stained with toluidine blue and cover-slipped in Permount mounting medium. Light micrographs were taken with a Leica DM5000 B research light microscope using a 100X objective and a Leica DFC 350 FX digital camera with Leica Application Suite V3 software. Images were transferred to PhotoShop for post-acquisition processing.

### Western Blots

Proteins from whole adult flies were prepared in lysis buffer: 25 mM Tris-HCL pH 7.5, 150 mM NaCl, 1% Triton X100, Roche protease inhibitors 1 tablet per 50 ml, ES56 5 µg/µl pepstatin 50 µM, and 2 mM indoleacetic acid. A single whole fly was transferred into 100 µl lysis buffer and frozen immediately. Samples were sonicated twice for 10 seconds, then centrifuged at 16 krpm at 4°C for 6 minutes. Cleared supernantant was used for Western blot analysis. Drosophila GGA antibodies were kindly provided by Drs. J. Hirst and S. Kametaka. Cathepsin L antibody was purchased from R&D Systems, Inc. Cathepsin D antibody was kindly provided by Dr. S. Kametaka. HRP-conjugated goat anti-rabbit antibody and goat anti-mouse antibodies were purchased from Millipore.

### Analyze *Bchs* Overexpression Eye Phenotype

Virgin females of *yw, OreR, GGA^P1^* and *GGA^Δ^* lines were crossed with *Bchs* overexpression line *GMR-Gal4 EP(2L)2299.* Sons were collected and aged for three days before dissection. For each replicate, 10 fly heads were cut between eyes and placed in 1 ml acidified ethanol (pH 2) for 24 hours. Absorbance measurements on four replicates were taken at wavelength 480 nm.

### Chloroquine Diet Experiments

#### LAMP-1-GFP stability assay

*yw; GMR-Gal4 UAS-LAMP1-GFP/CyO* flies were raised to third instar stage on standard Drosophila medium. Early third instar larvae were transferred to vials containing 2 g. Formula 4–24 Instant Drosophila medium (Carolina Biological Supply) reconstituted either with 6 ml 10 mM chloroquine (Sigma-Aldrich Co), 0.3% Proprionic acid, and 0.3% Tegosept (chloroquine supplement) or 6 ml 0.3% Proprionic acid, and 0.3% Tegosept (control). Larvae were aged for 36 h at 25°C, then the salivary glands were dissected and analyzed by fluorescence microscopy.

#### Chloroquine survival curves

*GGA^P1^* and *GGA^Δ^* flies were crossed with *yw* flies as described in the text, to generate knockout or control males, depending on the sex of the parents. As adults eclosed, adult male flies were immediately transferred onto chloroquine-containing media, which consists of 2 g. instant Drosophila media (Carolina Biological Supply Company) reconstituted with 6 ml of 20 mM chloroquine (Sigma-Aldrich Co), 0.3% Proprionic acid, and 0.3% Tegosept. The number of surviving flies was recorded daily.

#### Starvation Test

Flies were raised on normal fly food. After pupation, pupae were transferred to amino acid deprived food (3% agar, 5% sucrose, 0.3% Tegoset, and 0.3% Proprionic acid in PBS) [48]. After adults eclosed, adult males were collected within 6 hours time and transferred to fresh amino acid deprived food. The number of survivors was recorded daily.
